# Transcriptional Analysis of Apoptotic Cerebellar Granule Neurons Following Rescue by Gastric Inhibitory Polypeptide

**DOI:** 10.3390/ijms15045596

**Published:** 2014-04-01

**Authors:** Barbara Maino, Maria Teresa Ciotti, Pietro Calissano, Sebastiano Cavallaro

**Affiliations:** 1 Functional Genomics Center, Institute of Neurological Sciences, Italian National Research Council, Via Paolo Gaifami 18, 95126 Catania, Italy; E-Mail: barbara.maino@functional-genomics.it; 2 European Brain Research Institute, Via del Fosso di Fiorano 64, 00143 Roma, Italy; E-Mails: mariateresa.ciotti@cnr.it (M.T.C.); p.calissano@ebri.it (P.C.)

**Keywords:** apoptosis, cerebellar granule neurons (CGNs), gastric inhibitory polypeptide (Gip), gene expression, rescue, survival, transcriptional program, pathway

## Abstract

Apoptosis triggered by exogenous or endogenous stimuli is a crucial phenomenon to determine the fate of neurons, both in physiological and in pathological conditions. Our previous study established that gastric inhibitory polypeptide (Gip) is a neurotrophic factor capable of preventing apoptosis of cerebellar granule neurons (CGNs), during its pre-commitment phase. In the present study, we conducted whole-genome expression profiling to obtain a comprehensive view of the transcriptional program underlying the rescue effect of Gip in CGNs. By using DNA microarray technology, we identified 65 genes, we named *survival related genes*, whose expression is significantly de-regulated following Gip treatment. The expression levels of six transcripts were confirmed by real-time quantitative polymerase chain reaction. The proteins encoded by the *survival related genes* are functionally grouped in the following categories: signal transduction, transcription, cell cycle, chromatin remodeling, cell death, antioxidant activity, ubiquitination, metabolism and cytoskeletal organization. Our data outline that Gip supports CGNs rescue via a molecular framework, orchestrated by a wide spectrum of gene actors, which propagate survival signals and support neuronal viability.

## 1. Introduction

Neuronal apoptosis represents a distinctive mode of programmed cell death that is characterized by an intrinsic suicide program, by which a neuron orchestrates its own destruction. It is characterized by specific biochemical and morphological events, including fragmentation of nuclear DNA, breakdown of the cellular cytoskeleton, cell shrinkage and pyknosis, and the bulging out of the plasma membrane (blebbing) leading to the formation of apoptotic bodies [1,2]. During normal nervous system development, physiologically appropriate neuronal loss contributes to a sculpting process that removes approximately one-half of all neurons born during neurogenesis [3]. Neuronal loss subsequent to this developmental window is physiologically inappropriate for most systems and can contribute to neurological deficits, e.g., neurodegenerative diseases such as Alzheimer’s and Parkinson disease [2,4,5]. Elucidating the molecular mechanisms underlying neuronal apoptosis hence may contribute to our understanding of basic developmental biology and human neuropathology [6].

Cerebellar granule neurons (CGNs) represent, both *in vivo* and *in vitro*, a common model for the study of neuronal apoptosis [7–10]. Primary cultures of CGNs have been extensively utilized to examine the signal transduction mechanisms underlying neuronal apoptosis [11–13]. In this *in vitro* paradigm, CGNs undergo rapid apoptotic cell death within 24 h after removal of serum and lowering of extracellular potassium from 25 to 5 mM [8]. Apoptosis requires transcription and protein synthesis and becomes irreversible during the first six hours following induction. Before this “commitment point”, CGNs can be rescued by the activation of specific signal transduction pathways or by the treatment with specific neurotrophic factors [8,14–16]. Among these, we have recently showed that gastric inhibitory polypeptide (Gip) exerts a potent anti-apoptotic effect in cultured CGNs [16].

Gip is a 42-amino acid hormone, belonging to the glucagon superfamily of polypeptides and deriving from a 153-amino acid precursor [17–19]. In addition to its ability to regulate insulin secretion, Gip is known to exert a range of pleiotropic and extra-pancreatic actions [20–30]. Gip and its receptor show a widespread distribution in the central nervous system and have been implicated with neurogenesis, survival, synaptic plasticity and cognitive function [16,31–35]. The ability of Gip to regulate cell viability and apoptosis has been demonstrated in hippocampal and cerebellar granule neurons [16,36,37], as well as in β-cells [38–46] and osteoblasts [47]. Although the survival effects of Gip are initiated by a G-protein-coupled receptor and activate a variety of intracellular second messengers [48–50], these signaling pathways converge into the nucleus and regulate a transcriptional program governing cell life and death, which is still largely unknown. In this study we have used whole-genome expression analysis by DNA microarray technology to identify the complete spectrum of genes and pathways activated by Gip during the commitment phase of apoptosis of CGNs.

## 2. Results and Discussion

### 2.1. Whole-Genome Expression Changes Underlying Apoptosis Rescue by Gip

CGNs undergo apoptotic cell death after removal of serum and lowering of extracellular potassium from 25 to 5 mM [9] and can be rescued by Gip treatment. By using whole-genome oligonucleotide microarrays, we monitored whole gene expression profiles of CGNs six hours after induction of apoptosis or following rescue by a maximal effective dose (100 nM) of Gip.

**Figure 1.**
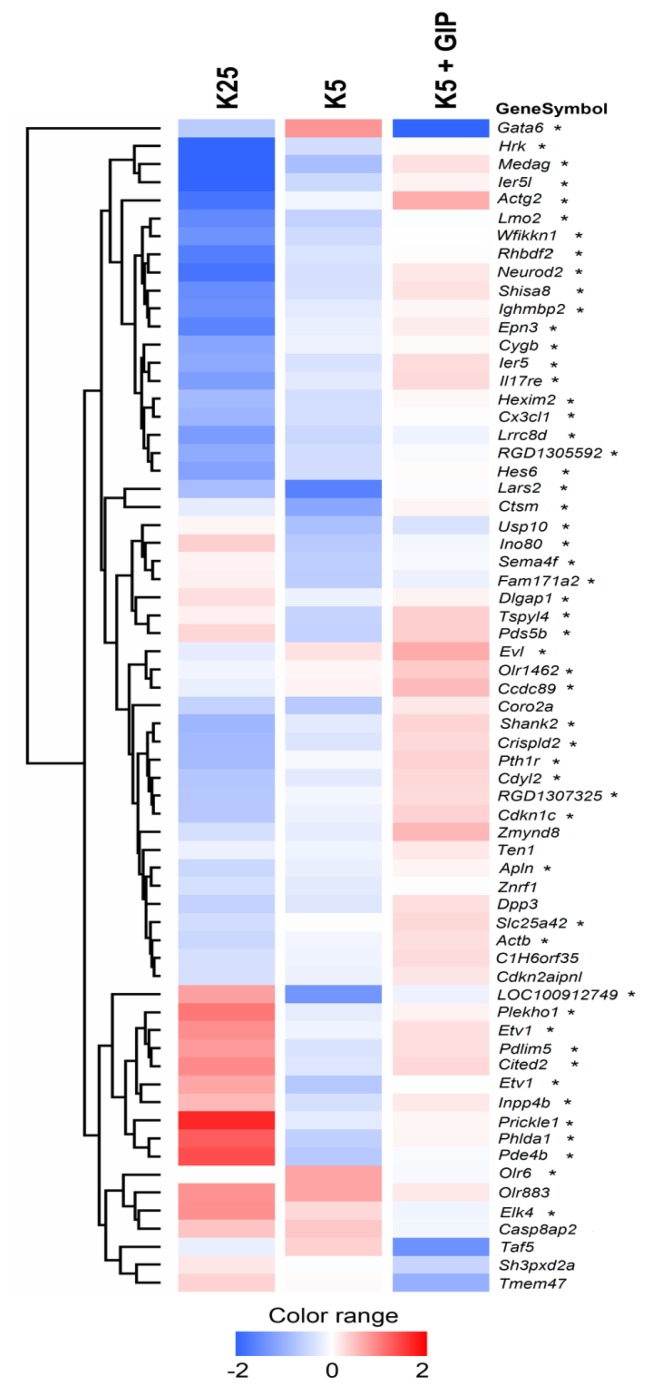
Hierarchical clustering of genes differently expressed in CGNs treated with Gip, during the pre-commitment of apoptosis. Sixty-five rescue genes (differentially expressed in K5 *vs.* K5 + Gip); were named using their UniGene symbol and were ordered into a dendrogram, whose length of the branches represents the relatedness of the expression levels in different experimental conditions. Data are presented in a matrix format: each row is equivalent to a single gene and each column is equivalent to one of the three different experimental conditions (K25, K5, K5 + Gip). The color of the corresponding cell in the matrix indicates the averaged normalized intensity from replicates. Red, blue and white respectively represent transcript levels below, equal or above the median abundance across all conditions. Color intensity reflects the magnitude of the deviation from the median (see scale below). Rescue genes differently expressed also following induction of apoptosis (K25 *vs.* K5) are indicated by *.

When gene expression profiles in *CGNs* 6 h after induction of apoptosis (K5) were compared to those of apoptosis rescued CGNs (K5 + GIP), 65 genes showed significant changes of gene expression. The majority (53/65) of genes differently expressed during rescue by Gip (K5 *vs.* K5 + Gip) were also differentially expressed following induction of apoptosis (K25 *vs.* K5). Figure 1 shows the gene expression patterns of rescue genes organized in a hierarchical cluster. Although our data represent the average gene expression from four replicates, we confirmed the reliability of the microarray data by real-time quantitative reverse transcription-polymerase chain reaction (RT-PCR). The expression pattern of four genes (*Egr1*, *Nipal2*, *Fam171a2*, *Nptx1*; differentially expressed in K5 or K5 + GIP) observed by microarrays closely paralleled the pattern observed using real-time PCR (Table 1).

**Figure 2.**
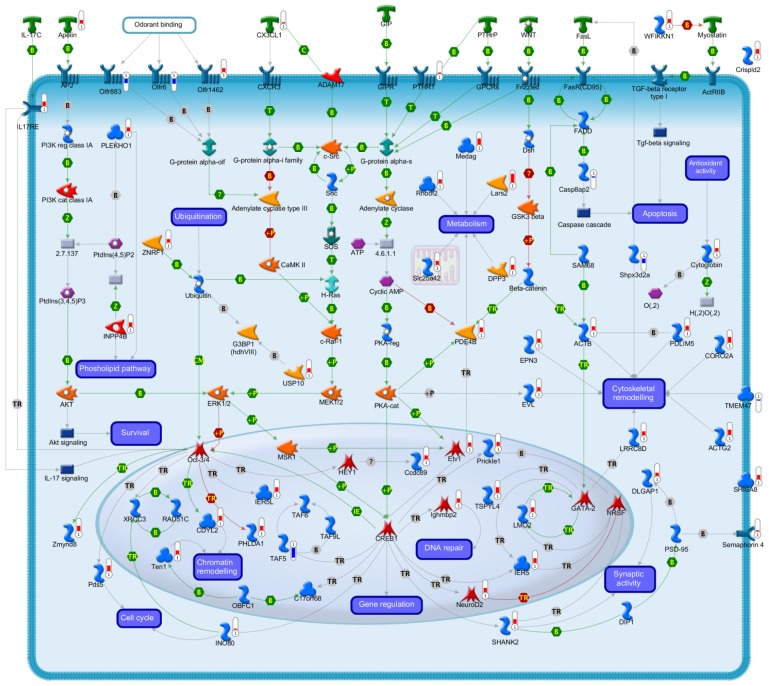

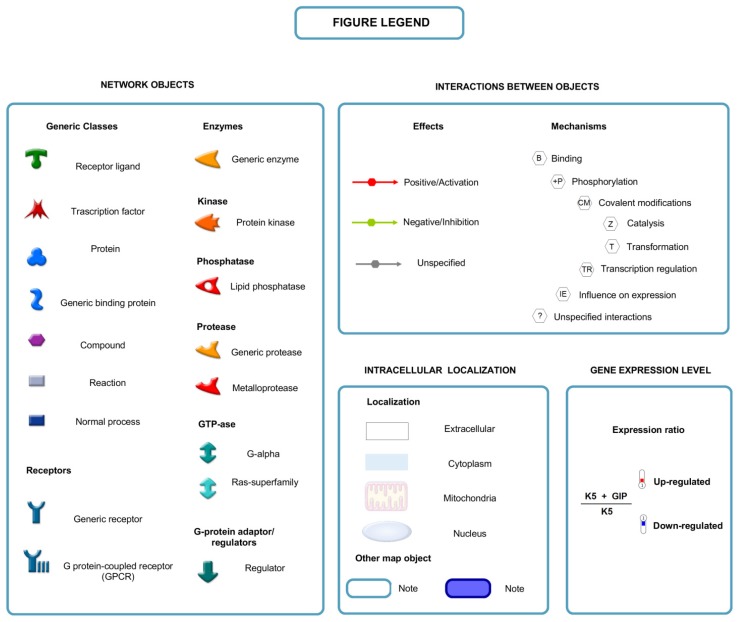
A comprehensive picture showing expression changes associated with CGNs rescue by Gip treatment. Fifty out of sixty-five “Survival related genes” (SGRs) encode proteins with a known function. This figure represents the sub-cellular compartments and molecular processes of these proteins. Each encoded protein is labeled with a thermometer that indicates gene expression changes: downward thermometers have a blue color and indicate down-regulated expression, whereas upward thermometers have a red color and indicate up-regulated expression. In addition to the SRGs, this Figure includes other genes (without a thermometer), which are part of a specific pathway. The figure legend below shows the set of symbols whereby network objects and interactions between objects are indicated in the figure. Mechanism of physical interactions between objects is indicated as follows: (**B**), binding; (+**P**), phosphorylation; (**Z**), catalysis; (**T**), transformation; (**TR**), transcription; (**CM**), covalent modifications; (**IE**), influence on expression; and (**?**), unspecified.

In the following paragraphs, transcriptional changes implicated in the present study will be discussed within the framework of deregulated gene groups and are represented in Figure 2.

### 2.2. Deregulated Gene Groups

#### 2.2.1. Signal Transduction

The most numerous group of differentially expressed genes, after treatment of CGNs with Gip, encodes proteins involved in signal transduction, which are mostly up-regulated. To facilitate understanding of how the neuroprotective effect of the Gip could be converted into appropriate cellular responses, we divided the translated products by this gene group into subcellular compartments, including extracellular, transmembrane, cytoplasmic and nuclear molecules (Figure 2).

The extracellular proteins include two neuropeptides, Apelin (Apln) and Chemokine C-X3-C motif ligand 1 (Cx3cl1), and two secretory proteins, Cysteine-rich secretory protein LCCL domain containing 2 (Crispld2) and WAP, follistatin/kazal, immunoglobulin, kunitz and netrin domain containing 1 (Wfikkn1), which are all up-regulated by Gip treatment.

Apln is a neuropeptide, known to prevent apoptosis in cortical neurons [51] and protect hippocampal neurons against NMDA receptor-mediated excitotoxicity [52–54]. Apln also enhances the protective effect of VEGF on H_2_O_2_-induced motor neuron death, whereas its deficiency accelerates motor neuron degeneration in the SOD1 (G93A) ALS mouse model [55]. Cx3cl1 is a neuron associated chemokine with neuroprotective effects in several *in vivo* and *in vitro* models of CNS pathology, including Parkinson’s disease [56–59]. Cx3cl1 attenuates excito-neurotoxicity and inhibits Fas-mediated cell death signaling [60–63]. Crispld2 is involved in neurogenesis, formation of normal neural crest cells and craniofacial development [64]. Wiffkn1 is a key inhibitor of Myostatin, which induces mitochondria-dependent apoptosis in the neuroblastoma cell line SH-SY5Y and slows muscle atrophy in the SOD1 (G93A) mice, an animal model of ALS [65–67].

A large group of transmembrane proteins is differentially expressed following Gip rescue. This group includes one over-expressed transmembrane adaptor protein, Shisa family member 8 (Shisa8), and six metabotropic receptors: Sema domain immunoglobulin domain transmembrane domain and short cytoplasmic domain (Sema4f), Interleukin 17 receptor E (Il-17re), Parathyroid hormone receptor 1 (Pthr1), Olfactory receptor 1462 (Olr1462) and Olfactory receptor 6 (Olr6) and Olfactory receptor 883 (Olr883). All of these metabotropic receptors are over-expressed, with the exception of Olr6 and Olr883.

Antagonizing Wnt and FGF signaling, Shisa8 is necessary for brain development [68,69], whereas interacting with Shisa9 it promotes short-term plasticity [69]. Sema4f, which is a member of the class IV subgroup of the semaphorin family, plays a role in neural development and glutamatergic synaptic plasticity in cultured hippocampal neurons [70,71]. The cytokine membrane receptor Il-17re exerts a great influence on adaptive immune cell recruitment in cases of CNS bacterial infection. High expression levels of Il-17re regulate the cellular mitogenesis via the RAS/MAPK signaling pathway, whereas a deficiency can induce CNS autoimmune disease [72–75]. Activation of the G-protein-coupled receptor Pthr1 elicits neuroprotective effects in rat cerebellar granule cells [76,77], regulating the l-type calcium channel and inhibiting kainic acid-induced excitotoxicity [78,79]. Similarly, Pthr1 expression in medulloblastoma protects from apoptosis [80]. Olr1462, Olr6 and Olr883 are members of the olfactory receptor family and as such play a role in the embryonic development of the human brain. Dysregulation of some cortical olfactory receptors has been associated with Parkinson disease [81].

Different genes, encoding for cytoplasmic proteins, are up-regulated following Gip treatment. This group includes two members of the phosphatidylinositol signaling pathway, Inositol polyphosphate-4-phosphatase type II (Inpp4b) and Pleckstrin homology domain containing, family O member 1 (Plekho1), one member of the type IV cyclic nucleotide phosphodiesterase (PDE) family, Phosphodiesterase 4B, cAMP-specific (Pde4b) and one serine and threonine-specific protein kinase Zinc finger, MYND-type containing 8 (Zmynd8). Inpp4b plays a neuro-proliferative effect against neurotoxicity induced by aluminium chloride AlCl_3_ in the experimental model of *Crocus sativus* L. [82]. High expression levels of Plekho1 appear to play a novel anti-apoptotic role against TNF reverse signaling in THP-1 and HEK293 cells [83]. Conversely low expression levels of Pde4b promote apoptosis in non-neuronal cells, such as the 3-D colonic-crypt [84] and B-cell model [85], as well as in the microglial cell line BV-2 [86]. In addition, deregulation in intracellular signaling mediated by PDE4B has been reported in schizophrenia and bipolar disorder [87]. Interacting with a member of the REST co-repressors group (RCOR2), Zmynd8 inhibits neural differentiation in Xenopus embryos [88].

It is interesting to note that a splice-variant of the Zmynd8 gene, called Protein kinase C-beta2 (PKCbeta2), regulates several cellular functions, including the inhibition of apoptosis [89].

In the cytoplasmic proteins subcategory, Gip induces two genes encoding synaptic proteins, SH3 and multiple ankyrin repeat domains 2 (Shank2) and Discs, large (Drosophila) homolog-associated protein 1 (Dlgap1) [90–93]. They both represent scaffolding proteins of excitatory synapses inside the CNS [93–95]. An increase in Shank2 expression and mutations of Shank2 have been reported in Alzheimer’s disease and in Plena McDermid Syndrome, respectively [96], whereas genetic variants in the Dlgap1 gene can induce schizophrenia [97].

Microarray screening of genes up-regulated in CGNs following Gip rescue highlighted four members of the nuclear proteins category: Prickle homolog 1 Drosophila (Prickle1), Pleckstrin homology-like domain, family A, member 1 (Phlda1), Coiled-coil domain containing 89 (Ccdc89) and Testis specific protein Y-linked 4 (Tspyl4).

Prickle1 is a nuclear receptor, which positively regulates planar cell polarity (PCP), both in CNS neurons [98] and in murine neuroblastoma (C1300) cells [99,100], and facilitates cortical neurogenesis [98]. Depletion in Prickle1 expression has been reported in human progressive myoclonus epilepsy (PME) [98,101]. Phlda1 (Tdag51) is a member of the pleckstrin homology-like domain family and although its anti-apoptotic role has not yet been found in neuronal cells, its protective effect has been shown in NWTb3 fibroblast mouse [102] and in the oral cancer cell line Ca9-22 [103]. Moreover, deficiency of Phlda1 expression promotes oxidative stress induced-apoptosis in MEFs, a mouse model of embryonic fibroblasts [104]. In contrast, high expression levels of Phlda1 are correlated with the evolution of intractable epilepsy (IE) [105]. Ccdc89 is a potential regulator of neurogenesis, interacting with the orange domain of the hairy-related transcription factor (HRT/Hey1) in *Xenopus* and mouse [106]. The nuclear protein Tspy4 is a member of the Tspy (testis-specific protein Y-encoded) family and, as such, is a sperm-specific biomarker in human cervico-vaginal fluids [107]. However the recent discovery that Tspy1 is found together with the multi-domain adapter protein CASK in neuronal axon fibers in the brain [108] could be the starting point to further investigate the role of Tspy4 in the development of the brain.

#### 2.2.2. Transcription

Microarray analysis showed that the Gip rescue regulates the expression of a considerable fraction of genes encoding proteins involved in transcriptional regulation, which can be divided into immediate-early and secondary-response genes.

The first subgroup includes the up-regulated *Immediate early response 5* (Ier5) and *Immediate early response 5-like* (Ier5l). Ier5 regulates the cellular response to mitogenic signals [109]. Its expression increases in rat cerebral cortex after sleep deprivation or spontaneous waking [110] and may also be considered a regulator of circadian rhythms. Ier5l is related to cellular and embryonic development [111].

The secondary response genes subgroup includes three transcription factors, Neuronal differentiation 2 (NeuroD2), Ets variant gene 1 (*Etv1*), Taf5 RNA polymerase II TATA box binding protein (TBP)-associated factor, 100 kDa (Taf5), and two transcriptional regulators, Immunoglobulin helicase μ-binding protein 2 (Ighmbp2) and *LIM domain only 2* (*rhombotin-like 1*) (Lmo2). All of these molecules are over-expressed by Gip treatment, with the exception of Taf5.

NeuroD2 is a basic helix-loop-helix (bHLH) protein, a member of the NeuroD family, which has pleiotropic functions, including neurogenesis, neuronal differentiation, development, connectivity and synaptic maturation [112–117]. Its over-expression, in particular, has been related to the survival of cerebellar and hippocampal neurons [118]. Deficiency of NeuroD2 in mice has been linked to amygdala dysfunction [116] and ataxia [117,118]. Similarly, a member of the ETS (E twenty-six) family, Etv1, controls a broad array of neuronal processes, including dopamine neuronal differentiation, maturation of granule cells by means of gene regulation (Nrc2, Tiam2 genes and more others) and Bdnf cascade signaling [119–122]. Etv1 induction also promotes sensory motor-circuitry [123], whereas Etv1 deficiency has been reported in Spinal muscular atrophy [124]. Although the Etv1 anti-apoptotic role in neurons has not been previously reported, its protective role has been demonstrated in the human breast cancer cell line MDA-MB-23 [125]. Taf5 is an integral subunit of the TFIID transcription complex that is known to stabilize the interaction with Taf6 [126], a pro-apoptotic protein which controls p53-apoptosis in human cells [127]. Down-regulation of Taf5 following Gip treatment, therefore, may induce a rescue effect by interfering with Taf6.

The transcriptional regulator Ighmbp2, which is over-expressed by Gip treatment, is a member of the helicase superfamily. Its encoded protein regulates replication, recombination and repair processes [128]. Defects in DNA repair are very often involved in cell death and a lack of Ighmbp2 may underlie altered DNA repair. In addition, several mutations of Ighmbp2 have been reportedin Distal Spinal Muscolar Atrofy type 1 (DMSA) [129,130]. Lmo2 controls cerebellum and hippocampal development and plays a role in neurogenesis, interacting with the pro-survival factors Slc and Gata2 [131,132].

#### 2.2.3. Cell Cycle

The Gip neuroprotective effect regulates the expression of two genes involved in cell cycle checkpoints: *Pds5*, *regulator of cohesion maintenance*, *homolog B* (*S. cerevisiae*) (Pds5b) and *Ino80 complex subunit* (*Ino80*). These two genes are both up-regulated following Gip treatment. Pds5b is a cohesion protein, known to pair replicated sister chromatids during cohesion in the S phase of the cell cycle [133,134]. Precise missense mutations of Pds5b have been associated to apoptosis in *saccharomyces cerevisiae* during early meiosis [135] and with the onset of Cornelia de Lange Syndrome (CdLS) [136]. Ino80 is a member of the chromatin remodeler family, which plays an efficient role in DNA synthesis, S-phase progression and chromosome segregation during the normal cell division cycle [137]. In yeast, Ino80 enhances chromatin mobility in response to DNA damage, acting downstream from the checkpoint factor Mec [138], whereas it suppresses genome instability, modulating chromatin remodelling complexes and repairing DNA double-strand breaks via the expression of *Rad54B* and *XRCC3* genes [139].

#### 2.2.4. Chromatin Remodelling

Two genes encoding proteins involved in chromatin remodelling are up-regulated by Gip during rescue of CGNs apoptosis: *Chromodomain protein Y-like 2* (*Cdyl2*) and *TEN1 CST complex subunit* (*Ten1*).

Cdyl2 is a member of the chromodomain Y chromosome (CDY) family and plays several roles, including histone modification and genome imprinting [140]. Ten1 is a member of the trimeric CTS complex (telomere-capping complex), which is essential for genome stability [141–143]. Insufficient telomeric DNA length or deficiency of key telomere-associated factors is known to elicit a DNA damage response, resulting in cellular senescence or apoptosis [144].

#### 2.2.5. Cell Death

Microarray analysis showed down-regulation of *Caspase 8 associated protein 2* (*Casp8ap2*) following Gip treatment. This gene encodes a multifunctional protein of the cysteine proteases family, which orchestrates the formation of the death complex [145,146], interacting with caspase-8 and activating Fas-mediated apoptosis and histone mRNA 3′ end processing [147,148].

#### 2.2.6. Antioxidant Activity

Gip rescue was associated with the up-regulation of a gene, *Cytoglobin* (*Cygb*), involved in the antioxidant defense system. The protein encoded by this gene is a member of the globin molecule family with a double functional nature: antioxidant activity, inducing superoxide dismutase, and anti-apoptotic activity, inhibiting the apoptotic effects of caspase-2 and caspase-3 [149–152]. In particular, Cygb plays a protective role against hypoxia-ischemia (HI) in rat brains [151] and safeguards cerebellar cells against chronic exposure to carbon monoxide (CO) during pre- and post-natal development [153].

#### 2.2.7. Ubiquitination

During Gip rescue we observed the over-expression of two members of the CGNs ubiquitin-proteasome system: Zinc and ring finger 1 E3 ubiquitin protein ligase (Znrf1) and Ubiquitin specific peptidase 10 (Usp10).

Znrf1 is a member of the ZNRF-E3 ubiquitin ligase family, which plays an important role in neuronal development, transmission, plasticity and also neuritogenesis by interacting with tubulin and glutamine synthetase (GS) [154–156]. Relevant to the present study is the previous demonstration that up-regulation of the Znrf1 gene induces an anti-apoptotic effect in MOLT-4 leukemia cells [157]. Usp10 is an ubiquitin-specific protease, which contributes to the formation of the stress granules interacting with GTPase-activating protein SH3 domain binding protein 1 (G3BP1) [158]. Stress granules are essential sites for translating stress-inducible genes and for reducing reactive oxygen species production [159].

#### 2.2.8. Metabolism

Microarray analysis showed an over-expression of five genes, encoding respectively two members of the serine protease subgroup, Rhomboid 5 homolog 2 (Rhbdf2) and Dipeptidyl-peptidase 3 (Dpp3), one aminoacyl-tRNAsynthetase, Leucyl-tRNAsynthetase 2, mitochondrial (Lars2), one mitochondrial protein carrier, Solute carrier family 25 member 42 (Slc25a42) and one adipogenic protein, Mesenteric estrogen dependent adipogenesis (Medag).

Rhbdf2 is a catalytically inactive member of the rhomboid family, which was recently reported as a key regulator of the anti-apoptotic TNF-alpha convertase enzyme (Adam17 or Tace) [160,161] that activates the transcription factor NF-kappa B in rat cortical neuron cultures after exposure to oxygen-glucose and glutamate deprivation [160–162]. Dpp3 is a member of the S9B peptidase family that is known to inhibit four pro-apoptotic genes, BCL2-like 10 (apoptosis facilitator) (BCL2L10), Tumor necrosis factor (ligand) superfamily, member 10 (TNFSF10), Tumor necrosis factor receptor superfamily, member 25 (TNFRSF25) and Tumor necrosis factor (ligand) superfamily, member 8 (TNFSF8) [163]. Dpp3 also activates the antioxidant response element (ARE) in primary mouse-derived cortical neurons, inducing NF-E2-related factor 2 (NRF2) nuclear translocation and NAD(P)H: quinoneoxidoreductase 1 [164]. Lars2 is a nuclear gene encoding the enzyme catalyzing the aminoacylation of mitochondrial tRNA (Leu) and its over-expression may represent an additional mechanism whereby CGNs contrast the induction of apoptosis and the partial inactivation of vital tRNALeu molecules [165]. In addition, over-expression of Lars2 in the human brain is a hallmark of a mitochondrial DNA point mutation (3243A > G) and may have a pathophysiological role in bipolar disorder and schizophrenia [166]. Slc25a42 is a novel member of the mitochondrial carrier family for coenzyme A (CoA) and adenosine 3′,5′-diphosphate [167,168] and is involved in the Stanley syndrome and Amish microcephaly [169]. Medag promotes adipogenesis and glucose uptake, interacting with several mediators, such as the fatty acid transporter CD36 that is involved in the uptake of the apoptotic material [170,171].

#### 2.2.9. Cytoskeletal Organization

Following Gip treatment, we observed the differential expression of a number of genes whose encoded proteins are involved with cytoskeletal rearrangements, cell shape, and motility, as well as dendritic spines [172]. These genes segregate into three groups: cytoplasmic actin isoforms, actin-binding proteins and other manipulators of the cytoskeletal organization.

The group of cytoplasmic actin includes two genes over-expressed by Gip: *Actin beta* (Actb) and *Actin*, *gamma 2*, *smooth muscle*, *enteric* (Actg2). Actb is one of the six different actin isoforms in humans and regulates synaptic formation and neuronal plasticity, interacting with the RNA-binding protein Sam68 [173,174]. Actb also plays a neuroprotective effect in propriospinal neurons after axotomy and in the MCF-7 breast cancer cells following treatment with salicylic acid [175]. A deficit in Actb gene expression alters cytoplasmic actin dynamicity and clinically induces Baraitser-Winter pathology and fragile X tremor/ataxia syndrome [173,176]. Consistent with the functions of other actin isoforms, Actg2 regulates the maintenance of the cytoskeleton, including actin-based motility in neurons [177]. Induction of Actg2, in particular, was found to rescue cardiac alpha-actin-deficient mice [178], whereas Actg2 repression contributes to cell cycle arrest following cadmium treatment of human lung fibroblast [179].

The group of actin-binding proteins includes the over-expressed *Coronin*, *actin binding protein*, *2A* (Coro2a) and *Enah/Vasp-like* (Evl). Coro2a is a member of the WD repeat protein family, which regulates motile processes, neuronal actin organization and focal-adhesion turnover through the cofilin signaling pathway [180,181]. Evl is a member of the Ena/VASP protein family, which enhances axon guidance, lamellipodial and filopodial dynamics in migrating cells, specifically binding the Sema6A-1 [182–184].

The group of manipulators of the cytoskeletal organization includes *Leucine rich repeat containing 8 family*, *member D* (Lrrc8d), *PDZ and LIM domain 5* (Pdlim5), *Epsin 3* (Epn3), *Transmembrane protein 47* (Tmem47) and *SH3 and PX domains 2A* (Sh3pxd2a). All of these, with the exception of Sh3pxd2a, are over-expressed following Gip treatment.

Lrrc8d, a member of the leucine-rich repeat protein family, is involved in B-cell development [185] and regulates cell adhesion and cellular trafficking. Pdlim5 is a LIM domain protein, which is involved in cytoskeleton organization, synaptic development and plasticity [186]. Epn3, a member of the Epsin family, stimulates neurite outgrowth and brain development interacting with Epn1 [187]. Tmem47 is a member of the PMP22/EMP/claudin protein family [188], which is required in the formation and sealing capacity of cell junctions mostly in the kidney [189]. Up-regulation of Tmem47 inhibits neurite outgrowth and regulates neuronal differentiation [190]. The human TMEM47 is considered a likely candidate for X-linked mental retardation [188]. Sh3pxd2a is an adapter protein, which regulates the outgrowth of podosomes, a type of actin-rich structure involved in tumor invasion and in pro-apoptotic signals. Interacting with the metalloprotease ADAM12, Sh3pxd2a may confer susceptibility to late-onset Alzheimer’s disease [191–193].

## 3. Experimental Section

### 3.1. Materials

All the substances were obtained from Sigma Aldrich (Milano, Italy) unless otherwise specified.

### 3.2. Neuronal Cultures

CGNs were obtained from dissociated cerebella of eight-day-old Wistar rats (Charles River, Lecco, Italy) (P8) and cultured as previously described [194]. Cells were plated in basal Eagle’s medium supplemented with 10% fetal calf serum, 25 mM KCl, 2 mM glutamine and 100 μg/mL gentamycin in poly-l-lysine coated 24-well clusters (NUNC, VWR International PBI s.r.l., Milano, Italy). Granule neurons were plated at 0.5 × 106 cells/well in BME.

### 3.3. Microarray Experiments

After six days “*in vitro*” (DIV), extracellular KCl of CGNs was shifted from 25 to 5 mM for neuronal apoptotic death induction. After two washes with serum-free BME containing 5 mM KCl, neurons were incubated with the same medium for 6 h (K5), while control neurons were incubated with serum free medium supplemented with 25 mM KCl (K25). K5 neurons were also treated with a maximal effective dose of GIP. After six hours of incubation, total RNA was extracted with Trizol (Life Technologies, Monza, Italy) from four biological replicates (derived from the same litter) for each of the experimental conditions (K25, K5, K5 + GIP). RNA integrity was confirmed by using a RNA chip and a 2100 Bioanalyzer (Agilent Technologies, Milano, Italy) with the protocol outlined by the manufacturer. Complementary RNAs (cRNAs) labeled with Cy3-CTP were synthesized from 1 μg of total RNA of each sample using the Low RNA Input Fluorescent Linear Amplification Kit (Agilent Technologies, Milano, Italy) following the manufacturer’s protocol. Aliquots (750 ng) of Cy3 labeled cRNA targets were hybridized on Whole Rat Genome Oligo Microarrays (Agilent Technologies, Milano, Italy). Microarray hybridization and washing were performed using reagents and instruments (hybridization chambers and rotating oven) as indicated by the manufacturer. Microarrays were scanned at 5-μm resolution using a GenePix Personal 4100A microarray scanner and the GenePix Pro 6.0 acquisition and data-extraction software (Molecular Devices, Sunnyvale, CA, USA). Raw data were processed and analyzed with GeneSpringGX 12.5 (Agilent Technologies, Milano, Italy). To remove unreliable data, all genes from all samples were filtered for quality to include only probe data fulfilling all of the following criteria in all replicates of at least one out of four experimental conditions: the spot had <3% of saturated pixels at 532 nm; the spot was not flagged “bad”, “not found” or “absent”; the spot was detectable well above background (signal-to-noise ratios at 532 nm were >10). Filtering data by quality control criteria short-listed 29,892 genes as our complete data set, out of a total of 41,012 probes present on the microarray. Genes in our quality-filtered data set were screened by a one-way ANOVA using Welch’s *t*-test, followed by the Benjamini and Hochberg False Discovery Rate procedure as a multiple testing correction and the Tukey’s Post Hoc test. Genes with a corrected *p* value <0.05 were selected as differentially expressed genes.

### 3.4. Real Time Quantitative PCR

Following extraction, total RNA samples (three/experimental condition) were reverse transcribed with an oligo(dT)12–18 and SuperScript II Rnase H-reverse transcriptase (Life Technologies, Milano, Italy). Aliquots of cDNA (0.1 and 0.2 mg) and known amounts of external standard (purified PCR product, 102 to 108 copies) were amplified in parallel reactions using primers indicated in Table 1. To control for the integrity of RNA and for differences attributable to errors in experimental manipulation, mRNA levels of mouse ribosomal S18 and phosphoglycerate kinase 1 were measured in similar reactions. Each PCR reaction (final volume 20 mL) contained 0.5 mM of primers, 2.5 mM Mg^2+^ and 1× DNA SYBR Green master mix (Roche Diagnostics, Monza, Italy). PCR amplifications were performed with a Light-Cycler (Roche Diagnostics, Monza, Italy) using the following four cycle programs: (i) denaturation of cDNA (1 cycle: 95 °C for 1 min); (ii) amplification (40 cycles: 95 °C for 0 s, 57 °C for 5 s, 72 °C for 10 s); (iii) melting curve analysis (1 cycle: 95 °C for 0 s, 67 °C for 10 s, 95 °C for 0 s); (iv) cooling (1 cycle: 40 °C for 3 min). Temperature transition rate was 20 °C/s except for the third segment of the melting curve analysis where it was 0.2 °C/s. Fluorimeter gain value was 7. The sequence of forward and reverse gene specific primers is shown in Table 1. Real-time detection of fluorimetric intensity of SYBR Green I, indicating the amount of PCR product formed, was measured at the end of each elongation phase. Quantification was performed by comparing the fluorescence of PCR products of unknown concentration with the fluorescence of the external standards. For this analysis, fluorescence values measured in the log-linear phase of amplification were considered using the second derivative maximum method of the Light Cycler Data Analysis software (Roche Diagnostics, Monza, Italy). Specificity of PCR products obtained was characterized by melting curve analysis followed by gel electrophoresis and DNA sequencing.

## 4. Conclusions

In the last years, the advent of full genome sequencing and high-throughput technologies are revolutionizing our ability to decode the underlying mechanisms of neuronal apoptosis and survival by offering a new approach based on systems biology. In this study we report for the first time a whole-genome analysis of these processes in CGNs following rescue by Gip. The results reveal the existence of a previously unknown transcriptional program associated with neuronal survival. The exact role and functional relationships of the genes implicated by gene expression profiling are mostly unknown and will require further studies. Systematic characterization of expression patterns associated to different rescue factors and in distinct temporal domains will also provide a framework for interpreting the biological significance of the expression patterns observed. Genetic or pharmacological exploitation of potential targets will help to determine their cause-relationship and identify new clues for neuroprotective drugs.

## Figures and Tables

**Table 1.** Validation of microarray data by real-time quantitative RT-PCR. Real-time PCR was used to validate the change in gene expression detected by microarray and to support the survival effects of Gip on CGNs.

Name	Genbank	K25	K5	K5 + GIP	Forward primer	Reverse primer
Early growth response protein 1 (Egr1)	U75397	−0.94	0.11	1.58	5′-GTTGGAATGCTGTGGTTACC-3′	5′-GCCAAACAAGTCACTTTGTTTA-3′
		1475 ± 85	3019 ± 108	3361 ± 102		
NIPA-like domain containing 2 (Nipal2)	NM_001130559	−1.26	−0.22	0.71	5′-ACATGGAGAAGCAACCTCTG-3′	5′-CTCCGTAATTGTCAGCAGCT-3′
		667 ± 16	2011 ± 91	4065 ± 133		
Family with sequence similarity 171, member A2 (Fam171a2)	XM_001081512	0.11	−0.63	−1.58	5′-AGGACAACGTGTACCGCAAT-3′	5′-TGGGGATCAGGTTGAGGGAA-3′
		2877 ± 128	1136 ± 92	871 ± 65		
DEAD (Asp-Glu-Ala-Asp) box helicase 56 (Ddx56)	NM_0010042112	0.35	−0.53	0.18	5′-TCTTAGGCTGTCACCGACTT-3′	5′-ATTAGCCACTCTCACATCGC-3′
		2493 ± 106	163 ± 12	2166 ± 77		
Zinc finger protein 423 (Zfp423)	XM_001081512	−1.23	−0.27	0.59	5′-GAAGACAGGAACAGCGTGAC-3′	5′-GTCGTCATCACCATCTCCAG-3′
		277 ± 31	856 ± 35	3184 ± 69		
Neuronalpentraxin I (Nptx1)	NM_153735	−1.50	−0.39	1.14	5′-GGAGCTGAATGGTTACATGG-3′	5′-ATAAGTCCACTGCGCACAGA-3′
		781 ± 32	2630 ± 85	4502 ± 181		

Microarray (upper row): mean normalized value (Log scale); Quantitative RT-PCR (lower row): mean ± SEM of copies/100 pg RT-RNA.
